# Toward Chalcogenide Platform Infrared Sensor Dedicated to the In Situ Detection of Aromatic Hydrocarbons in Natural Waters via an Attenuated Total Reflection Spectroscopy Study

**DOI:** 10.3390/s21072449

**Published:** 2021-04-02

**Authors:** Marion Baillieul, Emeline Baudet, Karine Michel, Jonathan Moreau, Petr Němec, Kada Boukerma, Florent Colas, Joël Charrier, Bruno Bureau, Emmanuel Rinnert, Virginie Nazabal

**Affiliations:** 1 Institut des Sciences Chimiques de Rennes, UMR-CNRS 6226, Equipe Verres et Céramiques, Université de Rennes 1, 35042 Rennes, France; Marion.Baillieul@upce.cz (M.B.); emeline.baudet@univ-rennes1.fr (E.B.); bruno.bureau@univ-rennes1.fr (B.B.); 2 IFREMER, Centre Bretagne, Laboratoire Détection, Capteurs et Mesures, CS10070, 29280 Plouzané, France; jonathan.moreau@ifremer.fr (J.M.); kada.boukerma@ifremer.fr (K.B.); florent.colas@ifremer.fr (F.C.); emmanuel.rinnert@ifremer.fr (E.R.); 3 BRGM, Direction Eau, Environnement et Ecotechnologies, Unité Bio-Géochimie Environnementale et Qualité de l’Eau, 45060 Orléans, France; k.michel@brgm.fr; 4 Department of Graphic Arts and Photophysics, Faculty of Chemical Technology, University of Pardubice, Studentska 573, 53210 Pardubice, Czech Republic; petr.nemec@upce.cz; 5 FOTON-UMR-CNRS 6082, ENSSAT BP80518, 22305 Lannion, France; joel.charrier@univ-rennes1.fr

**Keywords:** optical infrared sensor, chalcogenide glasses, mid-infrared, mono-aromatic hydrocarbons, natural waters, BTEXs, PAHs

## Abstract

The objective of this study is to demonstrate the successful functionalization of the surface of a chalcogenide infrared waveguide with the ultimate goal of developing an infrared micro-sensor device. First, a polyisobutylene coating was selected by testing its physico-chemical compatibility with a Ge-Sb-Se selenide surface. To simulate the chalcogenide platform infrared sensor, the detection of benzene, toluene, and ortho-, meta- and para-xylenes was efficaciously performed using a polyisobutylene layer spin-coated on 1 and 2.5 µm co-sputtered selenide films of Ge_28_Sb_12_Se_60_ composition deposited on a zinc selenide prism used for attenuated total reflection spectroscopy. The thickness of the polymer coating was optimized by attenuated total reflection spectroscopy to achieve the highest possible attenuation of water absorption while maintaining the diffusion rate of the pollutant through the polymer film compatible with the targeted in situ analysis. Then, natural water, i.e., groundwater, wastewater, and seawater, was sampled for detection measurement by means of attenuated total reflection spectroscopy. This study is a valuable contribution concerning the functionalization by a hydrophobic polymer compatible with a chalcogenide optical sensor designed to operate in the mid-infrared spectral range to detect in situ organic molecules in natural water.

## 1. Introduction

The research in the field of photonics for chemical sensors to detect water pollutants is ecologically appealing [1]. The increase in the number of marketed chemical compounds has led to a growing demand for monitoring systems with high enough stability, large sensitivity, broad range of detection, a compact format, and low response times [2,3,4]. With absorption being related to the vibration of organic pollutant compounds in the mid-infrared (MIR) spectral range (2.5–25 µm), the optical chemical sensors have seen advances recently [5]. Currently, the detection of aromatic hydrocarbons in water is usually carried out using gas chromatography–mass spectroscopy (GC-MS) analysis, which has some disadvantages such as high cost, artifacts related to sample handling, and long analysis times [6,7]. On the other hand, the exploitation of attenuated total reflection (ATR) or evanescent wave infrared spectroscopies is a promising field for in situ sensing [8]. Promising first results for the detection of aromatic hydrocarbons were obtained using different internal reflection elements, such as a trapezoidal zinc selenide (ZnSe) prism [9,10,11], a silver halide fiber [9,12,13], or a single reflection diamond waveguide [14]. These demonstrations have paved the way for the development of thin-film sensing devices devoted to MIR chem/bio sensing and assay platforms.

Given the completion of the integration on a chip of photonic sensors operating in UV-visible or near-IR [15], a miniaturization approach can also be seriously considered in mid-IR sensing. The development of a micro-sensor based on thin-film waveguide allows the miniaturization of the photonic device. Multi-analyte detection with a low volume of analyte requires the integration of MIR photonic components and should benefit from a relative low-cost production with a view of mass production. The MIR micro-sensor can use the evanescent field for detection, i.e., the fraction of IR light that is outside the optical waveguide. This evanescent field is very sensitive to variations due to the presence of organic compounds such as absorption with a much higher efficiency than a conventional ATR system [16]. Recent progress has made possible to combine on the same chip an MIR light source (such as Quantum Cascade Lasers) and waveguides as well as a transducer and a detector [17,18,19,20].

**Figure 1:**
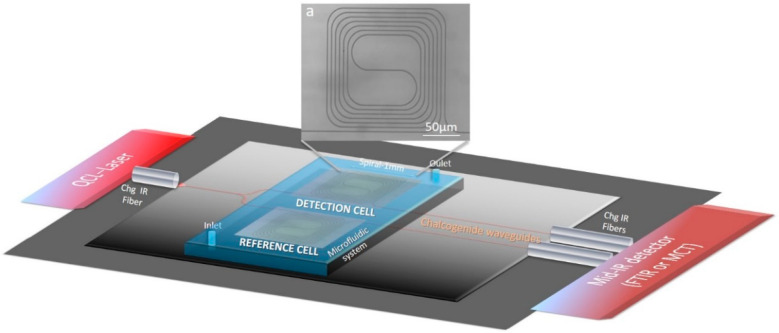
Mid-infrared (MIR) sensor scheme with MIR source, detectors, microfluidic system, and transductor made of Ge-Sb-Se waveguide functionalized with polyisobutylene (PIB), (**a**) SEM image of a single mode Ge-Sb-Se ridge waveguide in spiral configuration.

A large choice of waveguide material is available for a platform spectroscopy in the MIR [5,21]. Among them, chalcogenide thin films are suitable materials for many MIR photonics applications, including sensing [21,22,23,24,25,26,27,28,29,30]. They offer particular optical properties (broad window of transmission in the infrared and tailored refractive index), which make them interesting for integrated photonics devices. In previous papers, the authors reported the practicability of chalcogenide-based waveguides preparation targeting the MIR detection of some organic molecules, which can be present in water [16,30,31]. The first step toward the production of an integrated photonic sensor working in the MIR range was performed for Ge-Sb-Se selenide slab waveguide employing radio-frequency (RF) magnetron sputtering. Thus, single-mode selenide-based ridge waveguides in different configurations (for example spiral, Y-junction, S-bend) exploiting RF sputtering and reactive ion etching coupled with inductive conductive plasma process was designed and fabricated [31,32,33] (Figure 1a). Furthermore, the light injection efficiency experiment was performed in the MIR at 7.7 µm, and the light confinement was observed. Waveguide losses measurements resulted in encouraging values of ≈2.5 dB·cm^−1^ at this specific MIR wavelength. One of the important obstacles when detecting pollutant compounds dissolved in aqueous media is the heavy absorption of water in the MIR. In fact, it is almost impossible to detect aromatic hydrocarbons, even at high concentrations, when the internal reflection element is directly submerged in water [34]. The detection of pollutants using MIR spectroscopy requires surface functionalization of the optical component by a hydrophobic film. A membrane based on polymers is applied to extract organic compounds from water as well as to lower the volume of water along the beam. Various materials have been considered regarding their ability for molecules extraction, for example Teflon^®^ AF 2400 [10], poly(acrylonitrile-co-butadiene) (PAB) [10], polydimethylsiloxane (PDMS) [10], ethylene/propylene copolymer (EPco) [13,35,36], and polyisobutylene (PIB) [9]. Due to the good extraction of small aromatic hydrocarbons (benzene, toluene, or different xylenes, i.e., BTXs), the PIB has been firstly selected to functionalize Ge-Sb-Se chalcogenide thin-film waveguide to detect and quantify BTXs in water. The feasibility of deposition of PIB on a selenide ridge waveguide has already been demonstrated, as presented in Figure 1.

The objective of this study is to perform the functionalization of a selenide surface with the goal of developing a Ge-Sb-Se micro-sensor device [27,29] (Figure 1) for the in situ detection of selected organic compounds in real natural waters. First, the thickness of the PIB coating is adjusted experimentally to obtain the highest possible attenuation of water absorbance and at the same time maintain the diffusion rate of the pollutants through the PIB films adequate for in situ analysis. To simulate the selenide infrared waveguide, BTX detection is performed using PIB spin-coated on 1 and 2.5 µm Ge_28_Sb_12_Se_60_ co-sputtered films deposited on a ZnSe prism. The use of the ZnSe selenide prism is explained by the perfect compatibility in terms of refractive index and chemical composition with Ge-Sb-Se co-sputtered films in order to use, as a first approach, ATR spectroscopy for the convenience of detection before moving onto infrared spectroscopy on a selenide micro-sensor that is more complex to implement but allows a significantly increased sensitivity. This optimization of the selenide waveguide functionalization by a hydrophobic polymer is a crucial step contributing to the fabrication of the photonic micro-sensor working in the MIR spectral range to the detection of organic molecules in water. Then, to determine if pollutants can be detected in real aqueous matrices using this PIB layer with the characteristics selected to be potentially suitable for the selenide spiral waveguide (Figure 1), natural water is sampled for detection measurement. This study aims to detect aromatic hydrocarbons in several real natural complex waters with and without spiking: hydrocarbons polluted groundwater, water from a wastewater treatment plant, and seawater sampled above a shipwreck.

## 2. Experimental Methods

### 2.1. Chalcogenide Film Deposition by Co-Sputtering on ZnSe Prism for ATR Spectroscopy

**Figure 2:**
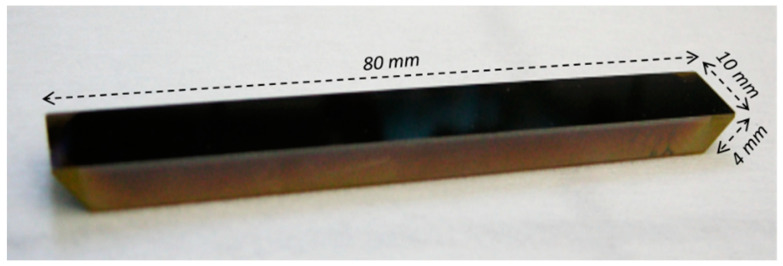
Ge_28_Sb_12_Se_60_ chalcogenide thin film deposited by RF co-sputtering on an attenuated total reflection (ATR) ZnSe prism.

Three trapezoidal ZnSe prism from PIKE Technologies (Madison, WI, USA) were used as MIR-transparent multi-reflection waveguides to propagate the signal and to detect the aromatic hydrocarbons using MIR-ATR spectroscopy. The dimensions of the ZnSe prisms were 80 × 10 × 4 mm, and the angle of incidence was 45°. A chalcogenide thin film was deposited on two ZnSe prism surfaces by radio-frequency (RF) magnetron co-sputtering from three commercial targets with the nominal composition Ge_28_Sb_12_Se_60_ (infrared chalcogenide glass IRG 25; Schott, Mainz, Germany). RF co-sputtering led to homogeneous and uniform chalcogenide thin films with 1.0 and 2.5 µm thicknesses on the whole ZnSe prism surface (Figure 2). The chalcogenide thin film was deposited at low RF sputtered power (due to the insulator nature of the targets) and at 10^−2^ mbar Ar pressure. The homogeneity, uniformity, thickness, and refractive index of chalcogenide films were characterized by ellipsometry in near infrared (NIR) and MIR wavelengths (total measured spectral range of 300–30,000 nm). For illustration, the refractive index values measured at 6.3 µm were similar on all prism surfaces (2.58 ± 0.01 and 2.55 ± 0.01 for prisms with 2.5 μm and 1 μm chalcogenide film thicknesses, respectively). The ZnSe prism of the lower refractive index (2.4245 at 6.33 µm) was used here for the excitation of propagation modes in the chalcogenide thin film. Simulations of the TE and TM propagation modes as well as the evanescent field intensity profile in the superstrate, i.e., in the PIB polymer film, were performed as a function of the thickness (1 μm and 2.5 μm) of the chalcogenide thin film and the index contrast of the selected materials at the BTX detection wavelength of 13.8 μm. It can be seen that for the two thicknesses considered of the Ge_28_Sb_12_Se_60_ thin film deposited on the surface of the ATR ZnSe crystal, single mode propagation is obtained to the extent that the IR beam from the ATR crystal is coupled into the chalcogenide layer. Thus, the intensity profiles (m = 0) of the evanescent field in the superstrate were determined for each of the two thicknesses of the Ge_28_Sb_12_Se_60_ layer. If we consider a chalcogenide layer deposited on the ZnSe prism, the evanescent field is much larger, which allows us to hope for a better detection of BTEX especially in the case of the thinner chalcogenide layer.

### 2.2. Polymer Coatings

Afterwards, a PIB polymer layer was deposited via spin coating (WS-400B-6NPP-Lite Spin Processor, Laurell Technologies, North Wales, PA, USA) onto the polished surface of the ZnSe prisms and the Ge_28_Sb_12_Se_60_ thin film deposited on the ZnSe prism. The polymer solution was prepared by dissolving polyisobutylene (PIB, Sigma Aldrich, St. Louis, MA, USA; M_w_ = 500,000 g/mol) in xylene (10% *w*/*v*) (Sigma Aldrich), and a 500 µL volume of the solution was put on the surface of the prism. To obtain different thicknesses of the polymer while maintaining the same viscosity of the liquid and avoiding multilayer deposition creating new interfaces [37], PIB was coated using rotational rates ranging from 400 to 650 rpm and then dried overnight at room temperature under a fume hood. The appropriate polymer thickness can be estimated considering the penetration depth (*d_p_*) of the evanescent field that emanates from the ZnSe prism (1).

(1) dp=λ2πn1[sin2θ−(n2n1)2]12

In Equation (1), *λ* is the wavelength of the incident light, *n*_1_ and *n*_2_ are the refractive indices of the prism (i.e., ZnSe) and the superstrate (i.e., PIB), respectively, and *θ* is the angle of incidence. In previous studies, it was concluded to have the polymer layer thickness roughly three times larger the penetration depth in order to get satisfactory sensitivity of detection as well as low response times [9,10]. Applied to the ZnSe prism, this formula gives a penetration length of approximately 1.22 μm at 1640 cm^−1^ [38]. Thus, in the range of 1640 cm^−1^ to 650 cm^−1^ corresponding to pollutants absorption, the penetration length considering a selenide waveguide and a PIB layer will vary approximately between 1.6 and 3.9 µm, respectively. It is necessary to obtain a compromise in order to avoid having a too thick layer limiting the response time of the infrared sensor but to prevent water absorption as much as possible in this spectral range. Therefore, this study will try to find this optimum to guarantee the efficiency of the future selenide micro-sensor. IR spectra were collected to confirm total solvent evaporation. The polymer thickness was calculated using a gravimetric technique [39] (2):

(2) $$L=\frac{m}{\rho A}$$

where *L* is the polymer layer thickness, *m* is the layer mass, *ρ* is the polymer density, and *A* is the coated surface area.

### 2.3. Water Sampling Sites

**Figure 3:**
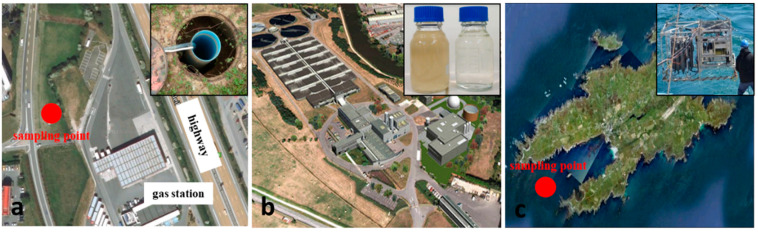
Water sampling sites. (**a**) Groundwater polluted by hydrocarbons near a gas station and a highway (south of Paris, France)—Inset shows the piezometer from which water was sampled; (**b**) wastewater treatment plant (Rennes, France)—Inset shows water samples from the input (left) and output (right) stations; (**c**) seawater sampled off the coast of Ushant Island (France) above the Peter Sif wreck—Inset shows profiler used to sample water.

Polluted waters were obtained from three different sites. The first site involved groundwater polluted by hydrocarbons. This site is located near an old gas station and a highway (south of Paris, France) (Figure 3a). Groundwater was sampled from two different piezometers. The second site involved wastewater that was collected from the wastewater treatment plant in Rennes (France); sampling was carried out in the input and output station (Figure 3b). In the third site, seawater was sampled off the coast of Ushant Island (France) above the *Peter Sif* wreck at two different depths (Figure 3c): the first at sea surface and the second at 30 m depth. All samples were collected in sealed glass bottles and stored in a cool and unlit place. An intercomparison approach was carried out using GC-MS or stir bar sorptive extraction/GC-MS (SBSE/GC-MS) analysis to determine the pollutant concentration.

### 2.4. BTX Solution Preparation

BTX stock solutions were prepared from compounds purchased from Sigma Aldrich diluted in methanol (Sigma Aldrich) at 50,000 mg/L for benzene, toluene, and xylenes. The xylene mixture contained ethylbenzene (16%, data from Sigma Aldrich analysis certificate). To obtain aqueous solutions in different concentrations, appropriate volumes of the stock solutions were diluted in water under stirring. The methanol concentration used as reference was fixed at 1% *v*/*v*. The BTX stock solution was used for spiking natural waters.

### 2.5. Fourier Transform Infrared Measurements

**Figure 4:**
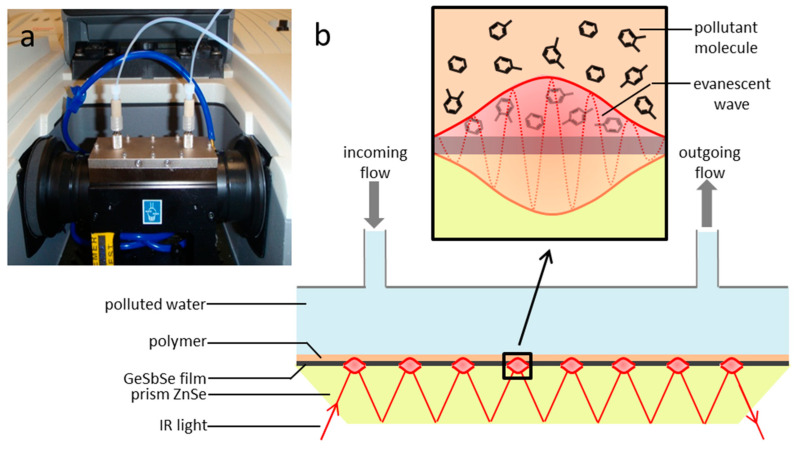
Image of ATR-Fourier transform infrared (FTIR) flow cell used for benzene, toluene, or different xylenes (BTX) detection (**a**)—Schematic representation of ATR-FTIR flow cell using a polymer (PIB)-coated GeSbSe planar waveguide deposited on ZnSe prism (**b**).

ATR-FTIR (Fourier transform infrared) experiments were performed using a Nicolet 6700 spectrometer equipped with a horizontal ATR accessory (PIKE Technologies), a ZnSe prism, and a fluidic flow cell. Aqueous solutions containing BTXs were brought over the functionalized surface of the ZnSe prism and GeSbSe:ZnSe prism with a peristaltic pump (Figure 4). Data were recorded within the spectral range of 400–4000 cm^−1^ using a spectral resolution of 2 cm^−1^. To avoid hydrocarbon losses caused by evaporation, all the solutions were freshly prepared and stored in quasi-hermetic glass bottles before and during the measurements. Spectral data were recorded every 10 min for 90 min. The characteristic bands for BTX determination are 674 cm^−1^ for benzene, 690 and 727 cm^−1^ for toluene, 741 cm^−1^ for ortho-xylene, 767 cm^−1^ for meta-xylene, and 794 cm^−1^ for para-xylene [40]. The characteristic infrared absorption of ethylbenzene, present in low amounts in the xylene mixture, is located at 696 cm^−1^, and thus overlaps the toluene peak and must be considered in this spectral range. These values are consistent with published values [36], which were determined using single-component enrichment experiments. Data are presented in terms of absorbance. To verify that the band intensity at each wavenumber is not affected by the wave depth penetration, intensities were also calculated using the *d_p_* relationship (1). This relationship depends on the wavelength and the refractive index of the medium (polymer, solution or atmosphere). No changes in trends were observed considering a constant PIB refractive index vs. wavelength in the IR domain. Thus, the ATR/IR intensities were not corrected.

### 2.6. Data Processing

The MatLab 7.0.1 algorithm was used to remove the spectral background in a consistent way for all the spectra while keeping the analytical signal intact [41]. To do so, the S function shown below was minimized:

(3) $$S=\sum_{\left(i\right)}\kappa_{i}{\left(y_{i}-z_{i}\right)}^{2}+\lambda \sum_{\left(i\right)}{\left(\Delta^{2}z_{i}\right)}^{2}.$$

In Equation (3), *y* is the signal intensity for each *i* wavenumber, *z* is the baseline, *λ* is the smoothing parameter, and *p* is the asymmetric parameter with $\kappa_{i}=p$ if *y_i_* > *z_i_* and $\kappa_{i}=1-p$ otherwise. The last term of Equation (3) is given in the following way:

(4) $${∆}^{2}z_{i}=\left(z_{i}-z_{i-1}\right)-\left(z_{i-1}-z_{i-2}\right)$$

The two variable parameters required for the calculations were set to *p* = 10^−2^ and *λ* = 10^5^. All spectra reported below underwent the same background correction. For the extraction and identification of the spectra, the SIMPLISMA algorithm was used [42], which was however modified for the automatic data processing in MatLab 7.0.1 [43]. The SIMPLISMA algorithm was able to extract the contribution of each compound. The relation exploited by SIMPLISMA is:

(5) D=CP+E

where *D* is the experimental data matrix, and C is the contribution matrix, which is proportional to the concentration. *P* is the variable matrix containing the pure spectral data and *E* is the residual error matrix. The determination of pure variables was stated within Equation (6).

(6) $$p_{i,k}=\left(w_{i,k}.\sigma_{i}\right)/\left(\mu_{i}+\alpha \right)\;$$

*p_i,k_* is the purity value of variable *i* from which the *k*_th_ pure variable is selected, *µ_i_* and *σ_i_* are the mean and standard deviation of variable *i*. α is an offset taking into account the noise range. The weight factor *w_i,k_* is a determinant-based function. It is corrected for previously chosen pure variables. The *Q* dispersion matrix was first calculated from *d*(*λ*). The data were scaled by the length to give an equal contribution for each variable. *d*(*λ*) is given by:

(7) d(λ)i,j= di,j/(μi2+(σi+α)2)12.

Then, the dispersion matrix was

(8) Q=(1n)D(λ)D(λ)T.

where *n* is the spectral number. The determinants were finally calculated according to Equation (9):

(9) $$w_{i,k}=\left|\begin{array}{c} \begin{array}{cc} Q_{i,i} & Q_{i,P1}\\ Q_{P1,i} & Q_{P1,P1} \end{array}\;\;\;\;\;\;\begin{array}{cc} \;\ldots & \;Q_{i,Pk-1}\\ \;\ldots & Q_{i,Pk-1} \end{array}\\ \begin{array}{cc} \ldots & \;\ldots \\ Q_{Pk-1,i} & \;\ldots \end{array}\;\;\;\;\;\;\;\;\begin{array}{cc} \;\;\;\;\;\ldots & \ldots \\ \;\;\;\;\;\ldots & Q_{Pk-1,Pk-1} \end{array} \end{array}\right|.$$

P_1_ is the first pure variable. The i index is the variable for which the determinant was calculated. The k index indicates the index of the pure variable for which this determinant was calculated. The relative contributions of the different species were obtained when the residual error delta was minimized according to Equation (10):

(10) $$∆\;=\;\sqrt{\frac{\sum_{i=1}^{n_{spec}}\sum_{j=1}^{n_{var}}{\left(d_{i,j}-d_{i,j}^{calc}\right)}^{2}}{\sum_{i=1}^{n_{spec}}\sum_{j=1}^{n_{var}}{d_{i,j}}^{2}}}.$$

In Equation (10), *d_i,_*_j_ is the *i^th^* row and *j^th^* column element of D; *d_i,j_^calc^* is the *i^th^* row and *j^th^* column element of D calculated by the SIMPLISMA algorithm; *n_spe_*_c_ is the number of the mixture spectra, and *n_var_* is the number of the recorded intensities. Some supplementary programming was carried out to automatically identify the different known chemicals based on their characteristic bands.

## 3. Results and Discussions

### 3.1. Optimization of the Polymer Coating

**Figure 5:**
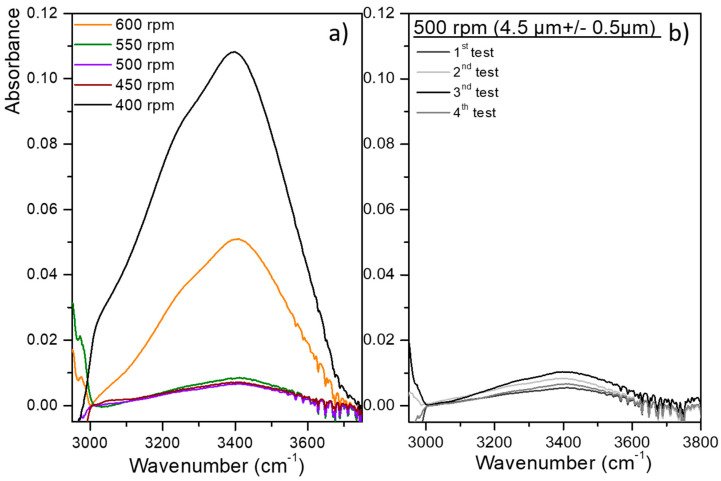
(**a**) MIR-ATR absorption spectra of water for different rotational rates (400–650 rpm)—(**b**) MIR-ATR spectra of water in the OH stretching range for four polymer layers (numbered from 1 to 4) deposited using the same deposition parameters, i.e., 500 rpm rotational rate, 500 µL volume of the solution, and rotational time of 30 s.

The optimization of the polymer coating was carried out to maximize the attenuation of the water absorption signal located between 3000 and 3700 cm^−1^, which are values that can be assigned to the O-H stretching vibration modes. Different thicknesses of the hydrophobic polymer coating were deposited by varying the spinner rotational rate on the ZnSe prism surface. All spin-coated depositions were performed with a constant rotational time set to 30 s. Figure 5a presents the MIR-ATR absorption water spectra collected after an enrichment time of 10 min and for different rotational rates (400–650 rpm). According to Equation (11) (with *t* representing the thickness of polymer, *η* representing the initial viscosity of the solution, *m* representing the solvent evaporation rate, *ρ*_0_ representing the initial mass concentration of solvent, and ω representing the rotational rate) [37], the thickness of the polymer layer increases with lowering the rotational rate. Two types of behavior were observed: (i) at low rotational rates (400 rpm), the attenuation of the water absorption signal is insufficient, even though the PIB thickness is expected to be the highest. In this case, the hydrophobic coating is probably inhomogeneous, i.e., with different thicknesses and even cracks across the prism surface, resulting in areas of strong water absorbance. (ii) At higher rotational rates (650–600 rpm), the PIB film thickness was lower around 2.5–3 μm (Equation (11)). Then, water is detected by the evanescent wave, although the PIB thickness is greater than the penetration depth at these wavelengths. According to these results, it seems necessary to have a thickness of the hydrophobic polymer that is much greater than the wave penetration of the medium when considering the wavelengths of water absorption without however risking being inhomogeneous when making a single thick film without making a multilayer to thicken the polymer, which generates other manufacturing constraints. Therefore, the optimum rotational rate allowing the higher attenuation of the water signal appears to be around 500 rpm depending on the experimental chemical conditions (PIB concentration and nature of the solvent).

(11) t=(3ηm2ρ0ω2)13

Afterwards, to validate the reproducibility of the polymer coating, four polymer layers (numbered from 1 to 4) were deposited using the same parameters, i.e., a rotational rate of 500 rpm. The attenuation of water signal was determined and thicknesses were calculated using the gravimetric technique (Figure 5b, Equation (2)). The thickness of the hydrophobic polymer coating was approximately 4.5 ± 0.5 µm for each deposition leading to a reproducible and suitable attenuation of water absorption signal. These results are consistent with the data found in the literature [10,34,36]. The thickness of the hydrophobic layer is sufficiently important that water absorption at 3000–3700 cm^−1^ and ~1640 cm^−1^ is weak because the evanescent field becomes negligible when the penetration depth (*d_p_*) is exceeded by 3 times (Equation (1)).

**Figure 6:**
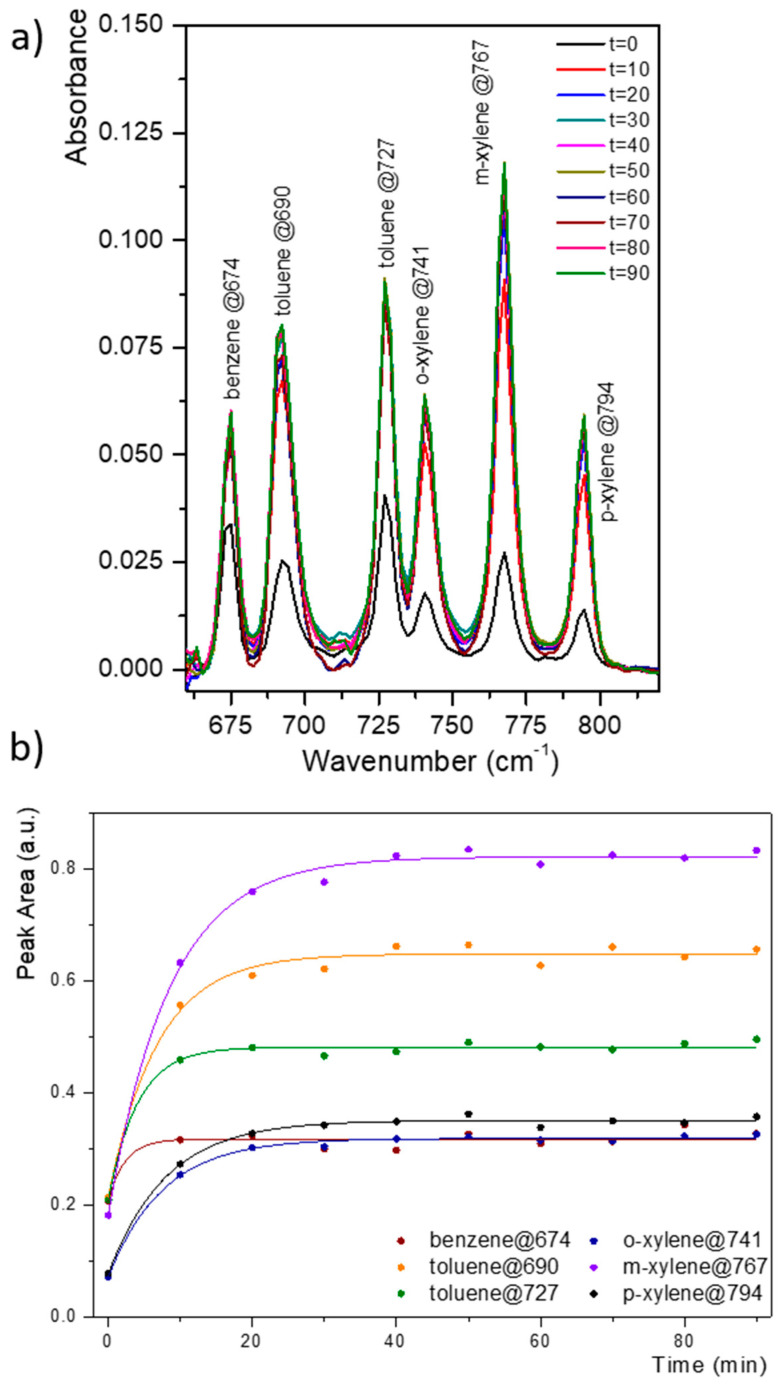
MIR-ATR absorption spectra of the BTX mixture (benzene, toluene, ortho-, meta-, and para-xylenes) at 5 ppm concentration obtained with PIB coated on a Ge_28_Sb_12_Se_60_ planar waveguide deposited on a ZnSe prism with a thickness of 2.5 µm (**a**)—Enrichment curves for the 5 ppm BTX (benzene, toluene, ortho-, meta- and para-xylenes) mixture obtained with a PIB coating on a chalcogenide thin film Ge_28_Sb_12_Se_60_ (t = 2.5 µm) on a ZnSe prism (**b**).

To simulate the chalcogenide optical waveguide of the IR micro-sensor, IR spectra of BTX mixtures were measured by means of a PIB coating on 1 and 2.5 µm Ge_28_Sb_12_Se_60_ thin films sputtered on a ZnSe prism. Figure 6a gives the MIR-ATR absorption spectra of the benzene, toluene, ortho-, meta- and para-xylenes mixture solution considering a concentration of 5 ppm at various times of detection. All aromatic hydrocarbons present in solution were simultaneously and immediately detected. Enrichment versus time curves for the BTX mixture are shown in Figure 6b; after 20–30 min of enrichment, a plateau was observed for each BTX. The diffusion rate of mono-aromatic hydrocarbons in the PIB polymer is comparable whatever the thickness of the selenide film composing the chalcogenide planar waveguide. Therefore, the development of an infrared micro-sensor composed of a spiral chalcogenide waveguide can be considered for mono-aromatic hydrocarbons such as BTX whose sensitivity could cover the range of tens ppb to hundreds ppm.

### 3.2. Analysis of Natural Waters—Groundwater

**Figure 7:**
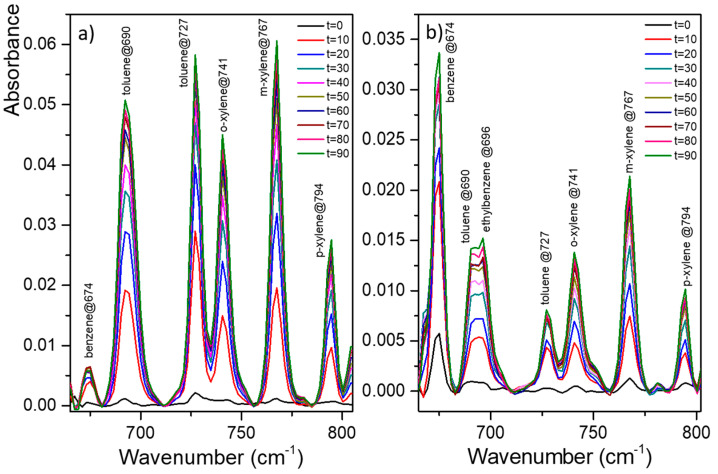
MIR-ATR absorption spectra of groundwater sampled in Piezometer 1 (**a**) and in Piezometer 2 (**b**) for different time enrichment (from 0 to 90 min) using a PIB coating.

**Figure 8:**
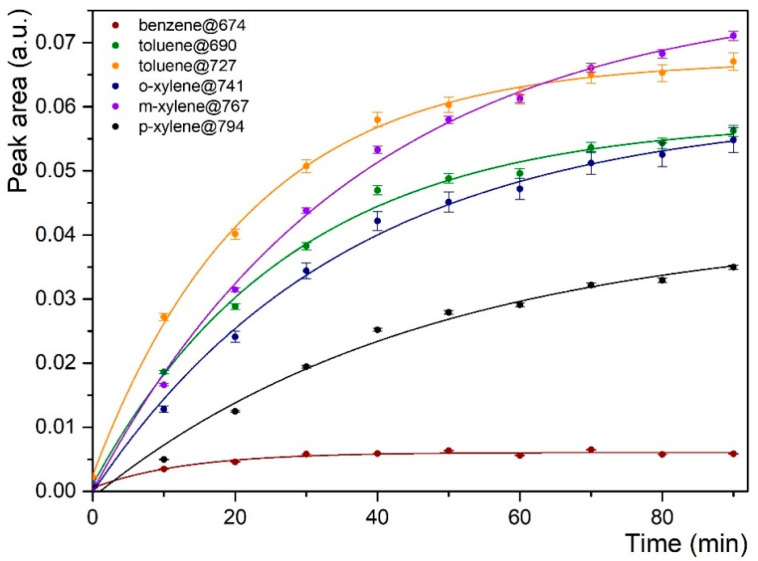
Enrichment curves for BTXs (benzene (1.7 ppm), toluene (18.2 ppm), ortho-, meta-, and para-xylene (25.97 ppm)) in sampled groundwater (Piezometer 1) obtained with PIB deposited on a ZnSe prism.

Detection experiments have been carried out on groundwater to prove the suitability of the PIB functionalization with natural complex water. For these measurements, the PIB polymer was deposited by drop casting onto the surface of the ZnSe prism. The drop-casted polymer may be thicker and more inhomogeneous than its spin-coated deposition counterpart. This was done in order to consider in situ analysis conditions where the regeneration of the coating would potentially be carried out directly on site and would not allow the use of a spin-coater. Therefore, the most drastic operating conditions of functionalization were tested for natural water. BTEX (E stands for ethylbenzene) concentration of groundwater was determined by GC-MS analysis, and the results of the two samples (Piezometers 1 and 2) are presented in Table 1. The MIR-ATR absorption spectra of groundwater sampled are given in Figure 7a (for Piezometer 1) and Figure 7b (for Piezometer 2). Hydrocarbon compounds in the groundwater were all detected as soon as the enrichment lasted 10 min, which is encouraging with the objective of a short response time for a field measurement (Figure 7a,b). The characteristic bands of BTEXs were located at 674, 690, 696, 727, 741, 767, and 794 cm^−1^ and were assigned to benzene, toluene, ethylbenzene, toluene, ortho-, meta-, and para-xylene, respectively. These results are in agreement with the BTX mixture previously studied. MIR-ATR absorption spectra of groundwater sampled in Piezometer 2 (Figure 7b) show a peak attributed to ethylbenzene. Despite the higher ethylbenzene concentration in water sampled in Piezometer 1, the compound was not observed in Figure 7a. Owing to the high concentration of toluene (i.e., 18.2 ppm), an overlap of toluene (at 690 cm^−1^) and ethylbenzene (at 696 cm^−1^) indeed interferes with molecule detection. Figure 8 presents the enrichment curve of BTXs in water sampled in Piezometer 1. In contrast to our previous experiment, a plateau was not reached, except for benzene that showed very low peak area values. In fact, the peak area for toluene and xylenes increased over time during the measurement. Moreover, although BTXs were detected using PIB deposited onto the ZnSe prism surface by drop casting, peak area values were much lower than the previous analysis with spin-coating deposition. Thus, owing to the drop-casting deposition of PIB, the hydrophobic layer is probably inhomogeneous, leading to slower diffusion of the pollutants in the polymer. The spin-coating deposition of PIB seems to allow a better diffusion and detection of hydrocarbons. It should also be kept in mind that this is a complex natural water that can also alter the diffusion of hydrocarbon molecules within the polymer regardless of its quality and will require further investigation.

### 3.3. Analysis Natural Water—Wastewater Treatment Plant

**Figure 9:**
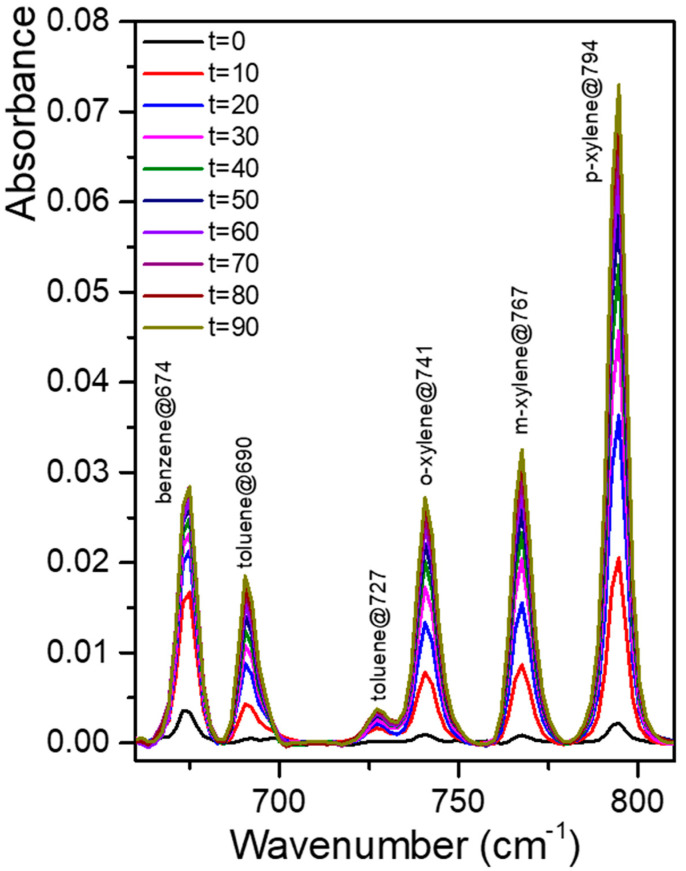
MIR-ATR absorption spectra of wastewater spiked with benzene (11.48 ppm), toluene (0.76 ppm), ortho-(3.08 ppm), meta-(6.04 ppm), and para-xylenes (11.88 ppm) for different time enrichment (from 0 to 90 min).

Wastewaters of the input and output station were analyzed using the ZnSe prism spin-coated with the PIB polymer. Based on previous results on groundwater, this time spin-coating was preferred for this complex water. GC-MS analysis was also performed in parallel. The wastewater did not contain any significant quantity of hydrocarbons or other organic pollutants, and no compounds were detected by ATR-FTIR spectroscopy. Thus, concentrations were below ppb levels. To check the appropriateness of the spin-coated PIB polymer layer for the detection of hydrocarbons in a complex matrix, BTXs were dissolved in input wastewater, which is the most complex matrix. Spiking concentrations of benzene, toluene, and ortho-, meta- and para-xylene are presented in Table 2. The MIR-ATR absorption spectra of wastewater spiked with BTXs are presented in Figure 9. The location of the peaks assigned to BTXs is in good accordance with those published in the literature [40] and our previous measurements; hydrocarbons were located at 674 cm^−1^ for benzene, 690 and 727 cm^−1^ for toluene, and at 741, 767, and 794 cm^−1^ for ortho-, meta-, and para-xylene, respectively. As soon as spiked wastewater was in contact with PIB, hydrocarbons were detected. Thus, the MIR-ATR system with spin-coated PIB allows a fast detection of BTX despite the complexity of the aqueous matrix and without any prior treatment of the wastewater.

### 3.4. Analysis Natural Water—Seawater

**Figure 10:**
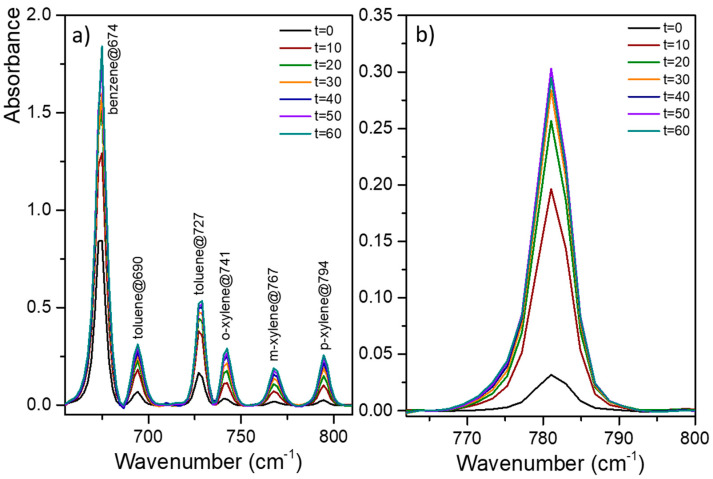
MIR-ATR absorption spectra of seawater (**a**) spiked with benzene (1790 ppm), toluene (530 ppm), ortho- (175 ppm), meta-, and para-xylenes (150/175 ppm) (BTXs) and (**b**) spiked with naphthalene, fluoranthene, and benzo[a]pyrene in the range of 17–18 ppm for different enrichment times (from 0 to 90 min for Seawater Sample 1 and from 0 to 60 min for Seawater Sample 2).

Finally, a third real natural water was analyzed using the spin-coated PIB functionalization. Two seawater samples were studied by MIR-ATR spectroscopy and SBSE/GC-MS. Similar to the wastewater analysis, there was no evidence of pollutants. The concentration of the molecules found in seawater was below ppt levels. Thus, seawater was spiked with BTXs (Seawater 1) and with polycyclic aromatic hydrocarbons (PAHs), i.e., naphthalene, fluoranthene, and benzo[a]pyrene (Seawater 2) that are more commonly found in contaminated seawater. Concentrations are given in Table 3. Figure 10a presents the MIR-ATR absorption spectra of Seawater 1 spiked with BTXs. The location of absorption peaks assigned to BTXs is in agreement with previous results. The detection of benzene, toluene, and xylenes was instantaneous: from t = 0, all spiking compounds were detected in the seawater sample. Likewise, Figure 10b shows the MIR-ATR absorption spectra of Seawater 2 spiked with PAHs. Peak absorption at 781 cm^−1^ was observed as soon as seawater was in contact with the polymer coating, i.e., t = 0 min. Peaks were assigned to naphthalene, due to its high concentration, with a possible overlap with fluoranthene and benzo[a]pyrene. Peak absorption at around 780 cm^−1^ is characteristic of =C-H out-of-plane bending modes in PAHs. To conclude, the MIR-ATR system using ZnSe prims spin-coated with PIB is promising for the detection of hydrocarbons in seawater; salinity nor the presence of organic matter in seawater do not interfere with measurements.

The detection limit obtained during this study using PIB coating directly on a ZnSe prism or on GeSbSe planar waveguide on a ZnSe prism for ATR-FTIR spectroscopy still needs to be improved, but it is of the same order of magnitude as other results found in the literature concerning the detection of hydrocarbons in aqueous media by means of infrared spectroscopy. Indeed, if detection techniques based on gas chromatography and microextraction allow detecting hydrocarbons at concentrations lower than 1 ppb [7,44,45], the detection of these molecules using ZnSe-ATR prism [11,36] and waveguide (planar [14], fiber [9,46]) in aqueous media (distilled and natural water) reaches limits of detection (LODs) ranging from a few tens to several hundreds of ppb. It is important to underline that the detection limit should be lower when using the chalcogenide waveguide, opening interesting perspectives for infrared microsensors based on the chalcogenide platform. For instance, a preliminary theoretical study aimed at detecting toluene molecules in aqueous media by an IR micro-sensor shows that the detection threshold will reach a value equal to 26 µg/L at λ = 6.66 μm [16,30], while by IR-ATR or IR fiber spectroscopy, an LOD of 60–339 µg/L was demonstrated for toluene [18,47].

## 4. Conclusions

In this work, the feasibility of an operational functionalization of the chalcogenide waveguide surface with the ultimate goal of developing an infrared micro-sensor device for the in situ detection of organic compounds in real natural waters was demonstrated. To lower the high water absorbance and to extract the polluting compounds from the solution, PIB was deposited by spin-coating on a ZnSe prism in order to deduce the optimum thickness, which corresponds to 4–5 μm. Then, to detect BTX in solution, MIR attenuated total reflection spectroscopy was performed using a ZnSe prism on which a selenide thin film of Ge_28_Sb_12_Se_60_ was deposited by RF magnetron co-sputtering to simulate the chalcogenide spiral waveguide of the final infrared micro-sensor. Different natural waters, i.e., groundwater, wastewater, and seawater, were analyzed. Despite the complexity of the aqueous matrices (turbidity, salinity, and organic matter), no interference was observed. In conclusion, the functionalization of planar Ge-Sb-Se selenide waveguides by a PIB layer seems perfectly adequate for the detection of pollutants in water, taking into account the optimized thickness of the PIB, the reproducibility, the uniformity and homogeneity of the PIB deposition obtained by spin-coating, the short response time (only a few minutes), and the non-influence of the complex matrix. These experiments confirm the feasibility of detection in real waters using a hydrophobic layer of PIB for the functionalization of the chalcogenide optical waveguide. Thus, the BTX detection obtained with PIB in this study should allow the development of an optical microsensor device based on chalcogenide films for in situ monitoring of pollutants in environmental waters.

## Figures and Tables

**Table 1:** BTEX (E stands for ethylbenzene) concentrations (ppm) of groundwater sampled in Piezometer 1 and Piezometer 2 measured by GC-MS.

	Benzene	Toluene	Ethylbenzene	Xylenes
Piezometer 1	1.70	18.20	6.16	25.97
Piezometer 2	12.90	2.46	2.25	6.85

**Table 2:** Benzene, toluene, ortho-, meta-, and para- xylenes (BTX) concentration (ppm) of the spiked wastewater determined by GC-MS.

	Benzene	Toluene	Ortho- Xylene	Meta- Xylene	Para- Xylene
Spiked wastewater	11.48	0.76	3.08	6.04	11.88

**Table 3:** Concentration (ppm) of seawater spiked with mono-aromatic hydrocarbons (benzene, toluene, ortho-, meta- and para-xylene) and spiked with polyaromatic hydrocarbons (naphthalene, fluoranthene and benzo[a]pyrene) measured by SBSE/GC-MS.

Spiked Seawater 1		Spiked Seawater 2	
benzene	1790	naphthalene	16.52
toluene	530	fluoranthene	0.52
ortho-xylene	175	benzo[a]pyrene	≤0.50
meta-xylene	150		
para-xylene	175		

## Data Availability

Not applicable.
